# Assessment of shape-based features ability to predict the ascending aortic aneurysm growth

**DOI:** 10.3389/fphys.2023.1125931

**Published:** 2023-03-06

**Authors:** Leonardo Geronzi, Pascal Haigron, Antonio Martinez, Kexin Yan, Michel Rochette, Aline Bel-Brunon, Jean Porterie, Siyu Lin, Diana Marcela Marin-Castrillon, Alain Lalande, Olivier Bouchot, Morgan Daniel, Pierre Escrig, Jacques Tomasi, Pier Paolo Valentini, Marco Evangelos Biancolini

**Affiliations:** ^1^ Department of Enterprise Engineering “Mario Lucertini”, University of Rome Tor Vergata, Rome, Italy; ^2^ Ansys France, Villeurbanne, France; ^3^ LTSI–UMR 1099, CHU Rennes, Inserm, University of Rennes, Rennes, France; ^4^ LaMCoS, Laboratoire de Mécanique des Contacts et des Structures, CNRS UMR5259, INSA Lyon, University of Lyon, Villeurbanne, France; ^5^ Cardiac Surgery Department, Rangueil University Hospital, Toulouse, France; ^6^ IMVIA Laboratory, University of Burgundy, Dijon, France; ^7^ Medical Imaging Department, University Hospital of Dijon, Dijon, France; ^8^ Department of Cardio-Vascular and Thoracic Surgery, University Hospital of Dijon, Dijon, France

**Keywords:** cardiovascular diseases, ascending aorta aneurysm, biomechanical features, classification, aorta, machine learning, growth rate, risk assessment

## Abstract

The current guidelines for the ascending aortic aneurysm (AsAA) treatment recommend surgery mainly according to the maximum diameter assessment. This criterion has already proven to be often inefficient in identifying patients at high risk of aneurysm growth and rupture. In this study, we propose a method to compute a set of local shape features that, in addition to the maximum diameter *D*, are intended to improve the classification performances for the ascending aortic aneurysm growth risk assessment. Apart from *D*, these are the ratio *DCR* between *D* and the length of the ascending aorta centerline, the ratio *EILR* between the length of the external and the internal lines and the tortuosity *T*. 50 patients with two 3D acquisitions at least 6 months apart were segmented and the growth rate (GR) with the shape features related to the first exam computed. The correlation between them has been investigated. After, the dataset was divided into two classes according to the growth rate value. We used six different classifiers with input data exclusively from the first exam to predict the class to which each patient belonged. A first classification was performed using only *D* and a second with all the shape features together. The performances have been evaluated by computing accuracy, sensitivity, specificity, area under the receiver operating characteristic curve (AUROC) and positive (negative) likelihood ratio LHR+ (LHR−). A positive correlation was observed between growth rate and *DCR* (*r* = 0.511, *p* = 1.3e-4) and between GR and *EILR* (*r* = 0.472, *p* = 2.7e-4). Overall, the classifiers based on the four metrics outperformed the same ones based only on *D*. Among the diameter-based classifiers, k-nearest neighbours (KNN) reported the best accuracy (86%), sensitivity (55.6%), AUROC (0.74), LHR+ (7.62) and LHR− (0.48). Concerning the classifiers based on the four shape features, we obtained the best accuracy (94%), sensitivity (66.7%), specificity (100%), AUROC (0.94), LHR+ (+*∞*) and LHR− (0.33) with support vector machine (SVM). This demonstrates how automatic shape features detection combined with risk classification criteria could be crucial in planning the follow-up of patients with ascending aortic aneurysm and in predicting the possible dangerous progression of the disease.

## 1 Introduction

Ascending aortic aneurysm (AsAA) is a risky dilatation of a weakened area of the ascending aorta (AAo) which may lead to dissection or rupture (Guo et al., 2018). Unfortunately, it is generally a silent pathology and the first symptoms may already indicate a serious and late-stage clinical situation with severe, life-threatening internal bleeding (Papagiannis, 2017). The estimated pooled incidence is between 5 and 10/100,000 individuals per year (Kuzmik et al., 2012; Melo et al., 2021). To date, the main criterion for elective ascending aortic surgery of non-urgent cases is the maximum diameter assessment whose decision threshold is generally fixed at 55 mm (Anfinogenova et al., 2022). Unfortunately, this does not seem to correctly reflect the AsAA patient’s risk of rupture (Elefteriades and Farkas, 2010; Tozzi et al., 2021) and is often considered insufficient as criterion of choice (Sonsino et al., 2022). In fact, many studies show aneurysms with diameters below the threshold for elective surgery which experience rupture while other aortas with huge diameters remain stable over time in terms of size (Smoljkić et al., 2017). Recently, the aneurysm growth rate (GR) (Oladokun et al., 2016) has also been included as a decision criterion for elective surgery. Therefore, as further stated in the clinical guidelines (Members et al., 2014), patients with rapid growth of the aortic diameter (more than 3 mm/year) should be considered for preventive surgical replacement. Furthermore, the process of aneurysm growth seems to be accelerated by the presence of a bicuspid aortic valve and congenital pathologies (Davies et al., 2007). For these reasons, the research is now focused on determining new biomarkers (Califf, 2018) for early diagnosis that can predict the aneurysm evolution and allow an accurate risk assessment. Some of those proposed have been associated to the shape of the aneurysm (Lindquist Liljeqvist et al., 2021) and mainly introduced for the abdominal aorta aneurysm (AAA). Among the measures related to the AAA shape, the vessel tortuosity and asymmetry seem to be highly relevant for predicting the rupture (Pappu et al., 2008; Doyle et al., 2009). According to Grobman and Stamilio (2006), the identification of local features on the shape of the abdominal aorta is valuable in assessing the risks of aneurysm rupture and establishing index thresholds for selecting patients to be surgically treated. An interesting framework describing the steps for a robust characterization of vascular geometries was proposed by Piccinelli et al. (2009) and applied to cerebral aneurysms. Here, all the steps from the image segmentation to the geometric characterization of the vascular structure are shown. The possibility of using algorithms to identify higher-risk patients has already been widely discussed (Saeyeldin et al., 2019). Shum et al., using a retrospective dataset of 76 patients, estimated geometric indices and regional variations in wall thickness and presented a decision tree algorithm to classify the data according to rupture criteria (Shum et al., 2011). Lee et al. (2013) proposed a classifier based on statistical machine learning for the curvature features of the abdominal aorta to evaluate the risk of rupture while Rengarajan et al. (2020) integrated biological information with geometric data to assess the same risk. Concerning the AsAA, the importance of estimating the length of the ascending tract for the decision of surgery has been demonstrated (Krüger et al., 2016; Wu et al., 2019). Kruger et al. presented a risk score based on the centerline length and on the maximum diameter (Krüger et al., 2018). Additionally, Poullis et al. (2008) showed how higher curvatures of the ascending aorta corresponded to higher forces exerted on the wall, explaining the potential effect this feature may have on the risk of aortic dissection. The AsAA risk of rupture has been assessed by considering also indices deriving from the ratio of the patient’s diameter and the height or body surface area (Zafar et al., 2018). Liang et al. (2017) presented a machine learning approach to evaluate a risk score for some patients previously tested with a structural simulation that brought the model to rupture. Jiang et al. (2020), using abdominal aortic aneurysm longitudinal data, Growth and Remodeling (G&R) techniques and Probabilistic Collocation Method (PCM), demonstrated how the diameter evolution over time can be better predicted using Deep Belief Network (DBN) compared to classical non-linear mixed-effect models (Sweeting and Thompson, 2012). Kim et al. (2022) used convolutional neural networks (CNNs) to predict the abdominal aortic aneurysm growth by integrating information of the vessel radius, thrombus thickness, Time Averaged Wall Shear Stress (TAWSS) derived from fluid-dynamic simulation and information of the exponential growth rate.

In this paper, we propose a method to obtain shape features to identify patients at high risk of AsAA growth. Besides the diameter, already proposed in the guidelines, these are the ratio between the diameter and the centerline length, the ratio between the length of the external and internal lines and the tortuosity of the ascending tract. Using longitudinal data derived from 50 patients, we segmented each image dataset to obtain a patient-specific geometry. After, we investigated the correlation between each shape feature computed on the first exam and the aneurysm growth rate calculated by exploiting the two acquisitions. Finally, using the shape features computed for the first exam only, we used and compared six different machine learning (ML) classifiers in order to predict the patients that could present adverse and fast AsAA evolution and show how these new local features can complement the information currently provided by the diameter alone.

## 2 Materials and methods

**FIGURE 1:**
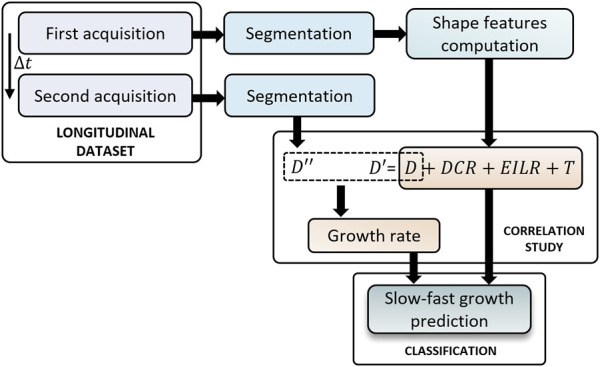
Full workflow for identifying patients at high risk of aneurysm growth.

The full pipeline of this study is presented in Figure 1. The current section is structured as follows: in Section 2.1 the dataset used for this study is described. In Section 2.2 we explain how the segmentation was performed while in Section 2.3 the methods for obtaining the shape features are presented. After, the method to compute the growth rate is reported in Section 2.4 and we conclude with Section 2.5 explaining the classification methods used to predict the risk class to which each patient belongs.

### 2.1 Data

To compute and evaluate the shape features, we used a retrospective dataset collected from the registry systems of three centers: the University Hospital of Rennes (Rennes, France), the University Hospital of Dijon (Dijon, France) and the University Hospital of Toulouse (Toulouse, France). The study has been conducted in accordance with ethical standards. The acquisitions were performed between December 2006 and September 2022 and the dataset was previously anonymized. Only the patients with at least two pre-operative 3D exams for which the official clinical report stated a condition of dilated aorta were included. We used both CT and MRI-Angiography images removing all the image dataset for which the resolution was worse than 1 mm × 1 mm × 1 mm. Additional exclusion criteria comprised 1) patients younger than 25 years, 2) prior valvular surgery, 3) aneurysms related to an infection or systemic inflammatory disease, 4) presence of acute aortic syndrome including dissection (type A, Stanford) and intramural hematoma, 5) diagnosis of congenital tissue disorders such as Marfan syndrome (Pyeritz and McKusick, 1979) and 6) existence of artefacts in the images. For each patient, we then considered the time interval between the two available acquisitions and 7) we excluded those with less than 6 months between the two longitudinal exams. Overall, 50 subjects, for a total of 100 exams, were included: 29 patients with a double ECG-gated acquisition, 21 with at least one non-gated. The longitudinal dataset included 86 (86%) CT-Scans and 14 (14%) MRI-Angiography. 72 acquisitions (72%) were performed with contrast agent injection and 28 (28%) without. The main characteristics of the patients, including the age at the date of the first acquisition, are given in Table 1.

**TABLE 1:** Characteristics of the dataset.

	Total = 50
Males (%)	33 (66%)
Age (in years)	58.1 **±** 13.2
Bicuspid valve	20 (40%)

### 2.2 Segmentation

For each patient, the segmentation of the whole thoracic aorta, from the aortic annulus to the descending aorta at the level of the diaphragm, was obtained using 3DSlicer (Fedorov et al., 2012). CT-Scans and MRI images were initially segmented using a semi-automatic local thresholding method (Senthilkumaran and Vaithegi, 2016) based on the grey level histogram derived from the analysis of three sets of voxels. Each set consisted of a different number of voxels (depending on the resolution of the images) distributed inside a sphere of radius 5 mm with the centre located at the middle of each of the three principal portions of the aorta: the ascending aorta, the aortic arch and the descending aorta. After extracting the 3-dimensional surface with Flying Edges algorithm (Schroeder et al., 2015), post-processing erosion and expansion methods were used to separate the structure of the aorta from other tissues and organs (Radl et al., 2022). The resulting surface underwent a manual editing process for verifying that it corresponded to the inner lumen of the vessel and for the correction of possible improperly segmented portions, especially in the case of geometries derived from MRI acquisitions. A median filter was then applied with a kernel size of 3 mm. At the end of the segmentation procedure, for each patient a tessellated surface made up of 8000–15000 triangular elements has been obtained.

### 2.3 Shape-based features

The geometric features we propose are all influenced by conformation, direction and length of the centerline (Dey and Zhao, 2002). It was extracted using the Vascular Modeling Toolkit (VMTK) through Voronoi diagrams (WSCG, 2003) after an automatic detection of the inlet and outlets seed points (Saitta et al., 2022). The segmented domain *S* related to the AAo and the corresponding centerline tract *C* were isolated with a first cut perpendicular to *C* at the level of the annulus and a second one in correspondence to the ostium of the brachiocephalic trunk. The methods to derive each shape feature are detailed below.

#### 2.3.1 Diameter

**FIGURE 2:**
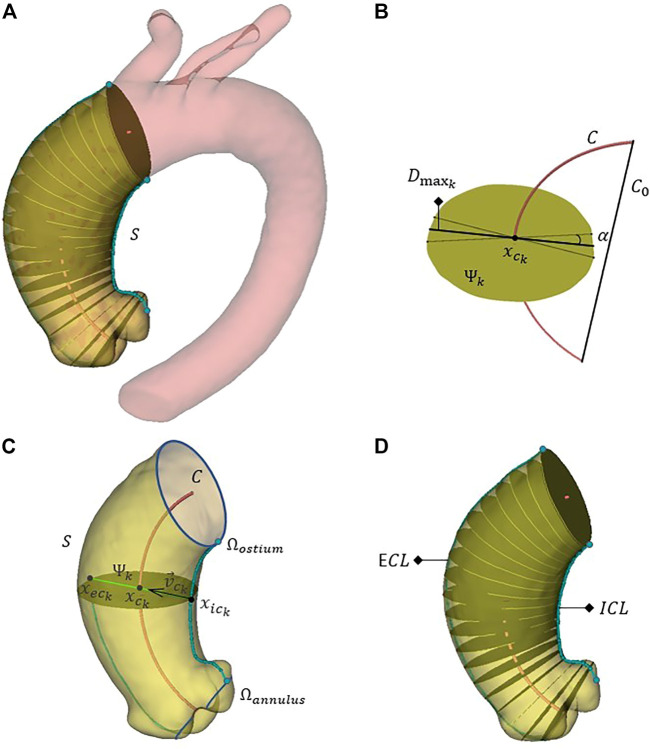
The full aorta segmentation, the discrete ascending aorta domain *S* and a subgroup of 20 aortic sections perpendicular to the centerline **(A)**. A generic section Ψ_*k*_ isolated for the calculation of the related maximum diameter $D_{\max_{k}}$ ; the centerline of the ascending section *C* and the segment *C*_0_ are also shown **(B)**. Identification of the point $x_{ec_{k}}$ to compute the external curvature line **(C)**. Isolated ascending section with the external and internal curvature line (*ECL* and *ICL*) **(D)**.

The AAo intra-luminal diameter is measured by extracting *n* = 100 sections Ψ_*k*_ for *k* = 1,…,*n* perpendicular to *C* and equally spaced along it. Figure 2A shows one of the thoracic aorta segmentations, the isolated ascending tract and a subset of 20 sections. On each section Ψ_*k*_, the maximum diameter $D_{\max_{k}}$ is obtained as the longest of the segments resulting from the intersection between Ψ_*k*_ itself and a rotating a plane perpendicular to Ψ_*k*_, passing through $x_{c_{k}}$ and sweeping angles of *α* = 10° (Figure 2B). In turn, $x_{c_{k}}$ is the point of intersection between Ψ_*k*_ and *C*. The maximum diameter *D* for the entire vessel, current main criterion for elective AAo surgery, is:

$$D=\max\left\{D_{\max_{1}},\ldots ,D_{\max_{n}}\right\}$$ (1)

#### 2.3.2 Diameter-centerline ratio

By using the maximum diameter *D* and computing the length of AAo centerline L(C) , we define the diameter-centerline ratio *DCR*:

$$DCR=\frac{D}{L\left(C\right)}$$ (2)

#### 2.3.3 External-internal line ratio

Given the tessellated surface *S* consisting of a set *F* of triangular faces *F*_*i*_ such that any point *P* ∈ *S* lies in at least one triangle *F*_*i*_ ∈ *F*, we can identify a polygonal curve $\Gamma_{j}^{x_{a},x_{o}}$ on *S* starting from an arbitrary point *x*_*a*_ ∈ Ω_*annulus*_ and reaching another arbitrary point *x*_*o*_ ∈ Ω_*ostium*_ where Ω_*annulus*_ and Ω_*ostium*_ are respectively the boundaries of *S* at the level of the annulus and at the level of the ostium. The length $L\left(\Gamma_{j}^{x_{a},x_{o}}\right)$ is:

$$L\left(\Gamma_{j}^{x_{a},x_{o}}\right)=\underset{F_{i}\in F}{\sum }L\left(\Gamma_{j_{|F_{i}}}^{x_{a},x_{o}}\right)$$ (3)

where $L\left(\Gamma_{j_{|F_{i}}}^{x_{a},x_{o}}\right)$ is measured according to the euclidean distance. We define the shortest discrete geodesic *Γ*_*G*_ as the shortest path:

$$\Gamma_{G}=\underset{x_{a},x_{o},j}{\text{argmin}}\;L\left(\Gamma_{j}^{x_{a},x_{o}}\right)$$ (4)

The length $L\left(\Gamma_{G}\right)$ results to be the shortest geodesic distance. We use the Dijkstra method (Lanthier, 2000; Dijkstra, 2022) to find the set of discrete geodesic over the entire aortic domain connecting all points *x*_*a*_ ∈ Ω_*annulus*_ with *x*_*o*_ ∈ Ω_*ostium*_ and we select the shortest of them. The resulting broken line is then smoothed to obtain the aortic internal curvature line (*ICL*).

Afterwards, for each section Ψ_*k*_, defining $x_{ic_{k}}$ the point of intersection between Ψ_*k*_ and *ICL*, the direction given by the versor $\vec{v_{c_{k}}}$ pointing towards the centre of the aorta $x_{c_{k}}$ is identified:

$$\vec{v_{c_{k}}}=\frac{\vec{x_{ic_{k}}x_{c_{k}}}}{\left\|\vec{x_{ic_{k}}x_{c_{k}}}\right\|}$$ (5)

The intersection between the axis along the direction $\vec{v_{c_{k}}}$ and *S* defines a new point $x_{ec_{k}}$ (Figure 2C). The repetition of this procedure on the *n* sections Ψ_*k*_ allows to create a spline corresponding to the external curvature line (*ECL*). In Figure 2D, *ECL* and *ICL* are shown. The ratio between the external and internal curvature line lengths *EILR* is then computed:

$$EILR=\frac{L\left(ECL\right)}{L\left(ICL\right)}$$ (6)

#### 2.3.4 Tortuosity

The last local shape feature we compute is the tortuosity *T*, defined as:

$$T=\frac{L\left(C\right)}{L\left(C_{0}\right)}$$ (7)

where *C*_0_ is the straight line connecting the first and the last points of *C*.

Except for the manual identification of the brachio-cephalic trunk ostium, the procedure for computing the shape features is without any user interaction. The geometric decomposition methods are developed using Python, VTK, ITK and Qt in 3DSlicer environment.

### 2.4 Growth rate evaluation

Even if the diameter threshold for elective surgery has not yet been reached, it is however clear that a patient with rapid AsAA growth over time should be carefully and constantly monitored (Geisbüsch et al., 2014). In this regard, we assume that the risk of aneurysm rupture is intrinsically derived from the risk of aneurysm growth (Coady et al., 1997; Saliba et al., 2015). Exploiting longitudinal data, the AsAA GR can be evaluated as ratio between the difference in maximum diameters and the time interval Δ*t* in months between the two scans:

$$GR=\frac{D^{\prime \prime }-D^{\prime }}{\Delta t}$$ (8)

where *D*′ is the diameter *D* related to the first exam and *D* ^″^ to the second acquisition. The Mann-Whitney test is used to compare the GR values derived from ECG-gated data with those computed on patients for whom at least one acquisition was not gated. The relationship between the proposed local shape features and the growth rate is then evaluated using Spearman’s correlation coefficients. Statistical analysis is performed using Matlab (version 9.12.0, R2022a).

### 2.5 Machine learning classification

We divided the patients into two risk classes according to the observed growth rate. All patients with GR ≤ 0.25 mm/month composed the low-risk class (41 patients) while the others represented the group with rapid growth (9 patients). This threshold was chosen according to the surgery guidelines previously mentioned. For every individual, we then tried to predict the belonging class by using ML classifiers with in input the metrics derived from the first acquisition acting as possible predictors of growth. We initially tested the diameter *D* alone derived from the first exam in order to predict the GR-related risk class. Then, a second classification was conducted selecting all the shape features together. Six different classification models (Silva et al., 2019) were used: decision tree (DT) (Ali and Smith, 2006), linear discriminant (LD) (Duda et al., 2000), logistic regression (LR) (Dreiseitl and Ohno-Machado, 2002), naive bayes (Ren et al., 2009), support vector machine (SVM) (Cortes and Vapnik, 1995) and k-nearest neighbours (KNN) (Altman, 1992). Except for LR, the hyperparameter values were optimized minimizing the classification error. We use a leave-one-out cross-validation method to assess the predictive accuracy of the classification models. The accuracy is defined as:

$$accuracy=\frac{\text{TP }+\text{ TN}}{\text{TP }+\text{ TN }+\text{ FP }+\text{ FN}}$$ (9)

Sensitivity and specificity are calculated as:

$$sensitivity=\frac{TP}{TP+FN}$$ (10)

$$speci\mathrm{fi}city=\frac{TN}{TN+FP}$$ (11)

where true positive (TP) is the number of fast-growing aortas correctly identified, true negative (TN) the number of low-risk shapes correctly identified, false negative (FN) the number of high-risk geometries incorrectly identified as low risk and false positive (FP) the number of low-risk shapes incorrectly identified as high risk. We obtained these values analyzing the confusion matrix, a 2 × 2 matrix where the diagonal represents the aortas that were correctly classified and the anti-diagonal represented misclassifications. In addition to accuracy, sensitivity and specificity, the performances were measured using the area under the receiver operating characteristic (AUROC) curve which represents the probability that the input parameter (parameters) is (are) higher for the class with fast growth than for the one with slow growth and thus, is a measure of discrimination. Finally, to describe the diagnostic value of the proposed shape features, likelihood ratios (LHRs) are used:

$$LHR+=\frac{sensitivity}{1-speci\mathrm{fi}city}$$ (12)

$$LHR-=\frac{1-sensitivity}{speci\mathrm{fi}city}$$ (13)

LHR+ (LHR−) represents the change in the odds of having a diagnosis in patients with a positive (negative) test.

## 3 Results

The full dataset presented a mean follow-up of 31 ± 25 months. AAo segmentations were performed for all 100 acquisitions and the shape metrics were extracted for the first exam of all patients. The median GR was 0.09 mm/month with an interquartile range IQR = 0.17 mm/month, in agreement with what reported in (Coady et al., 1999; Adriaans et al., 2021). The highest derived growth rate was 0.56 mm/month. The null hypothesis that GRs computed from ECG-gated and non-gated acquisitions can be attributable to a distribution with equal median was accepted (*p* = 0.048).

**FIGURE 3:**
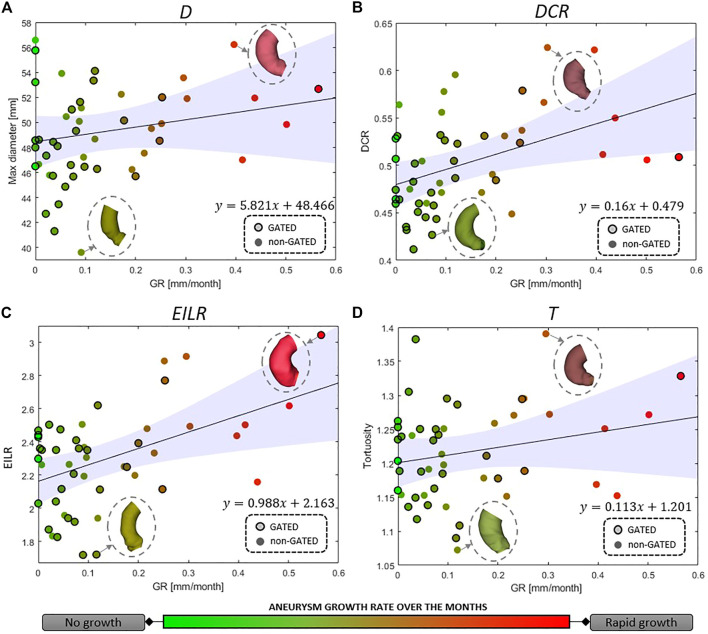
Correlation between growth rate and **(A)** maximum aneurysm diameter *D*, **(B)** ratio of maximum diameter and centerline length *DCR*, **(C)** ratio of external and internal curvature line length *EILR* and **(D)** tortuosity *T*. The circles around the marker edges indicate patients with two gated acquisitions in the same phase of the cardiac cycle. The line for linear correlation with 95% fitting confidence bounds is reported, the fitting equation provided and some of the AAo shapes shown.

For the full set of patients, the values of the computed shape features, expressed as median (IQR), are the following: 48.64 mm (5.46 mm) for *D*, 0.50 (0.07) for *DCR*, 2.32 (0.35) for *EILR* and 1.21 (0.10) for *T*. In Figure 3, we report the relationship between the shape features and the growth rate: the colour of each marker provides an idea of the GR “intensity,” from light green (no growth) to bright red (fast growth). A positive correlation is observed between GR and all four derived measurements. We obtain the following Spearman’s coefficients: *r* = 0.169 (*p* = 0.091) for *D*, *r* = 0.511 (*p* = 1.3e-4) for *DCR*, *r* = 0.472 (*p* = 2.7e-4) for *EILR*, *r* = 0.161 (*p* = 0.121) for *T*. A statistically significant moderate correlation between *DCR* and GR and between *EILR* and GR is thus evident while the shape features *D* and *T* do not reach the significance p-level.

**FIGURE 4:**
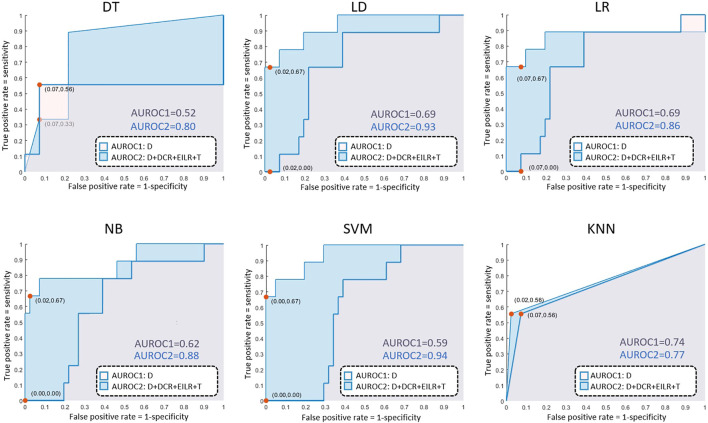
AUROC results for the decision tree (DT), linear discriminant (LD), logistic regression (LR), naive bayes (NB), support vector machine (SVM), k-nearest neighbours (KNN) classifiers.

**FIGURE 5:**
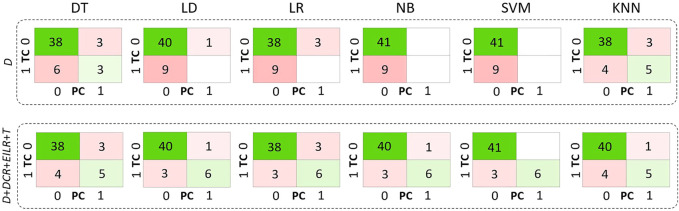
Confusion matrices related only to *D* on the first row and to *D*, *DCR*, *EILR* and *T* on the second row for the decision tree (DT), linear discriminant (LD), logistic regression (LR), naive bayes (NB), support vector machine (SVM), k-nearest neighbours (KNN) classifiers. TC means true class while PC means predicted class.

Concerning the classification, 9 patients (18% prevalence) were identified with growth rates above the threshold of 0.25 mm/month. The confusion matrices are reported in Figure 4 both for the diameter alone as growth predictor and for the four shape features together. Table 2 reports the performance of the six classifiers in terms of accuracy, sensitivity, specificity, LHR+ and LHR−. The AUROC for the six classifiers is shown in Figure 5. Using only *D* as risk class predictor, four of the classifiers (LD, LR, NB, and SVM) prove unable to identify high-risk patients (sensitivity = 0%). Among the *D*-based classifiers, KNN reports the highest accuracy (86%), sensitivity (55.6%), AUROC (0.74), LHR+ (7.62) and the lower LHR− (0.48). Instead, the KNN specificity is 92.7%, lower than that resulting from LD (97.6%), NB (100%), SVM (100%) and equal to the values obtained through DT and LR. On the other side, considering the four shape features together, the best performances are obtained using SVM. It returns the highest accuracy (94%), sensitivity (66.7%), specificity (100%), AUROC (0.94) and LHR+ (tending to *∞*). Even for LHR−, the best performance among the classifiers is achieved with a value of 0.33. In this case, DT is performing worse showing accuracy = 86%, sensitivity = 55.6%, specificity = 92.7%, LHR+ = 4.56, LHR− = 0.48 and AUROC = 0.52. Figure 5 shows how the AUROC is always superior for the classifiers with the four shape parameters as input compared to the equivalent diameter-only based classifiers. This is also clear by analyzing Table 2, where all the values calculated for the classification with the four parameters are equal to or outperform those of the classification with the diameter alone, except for the NB classifier specificity.

**TABLE 2:** Classification scores for the six classifiers obtained with leave-one-out cross-validation: decision tree (DT), linear discriminant (LD), logistic regression (LR), naive bayes (NB), support vector machine (SVM) and k-nearest neighbours (KNN).

	DT	LD	LR	NB	SVM	KNN
Accuracy (*D*)	82%	80%	76%	82%	82%	**86%**
Accuracy (*D* + *DCR* + *EILR* + *T*)	86%	92%	88%	92%	**94%**	90%
Sensitivity (*D*)	33.3%	0%	0%	0%	0%	**55.6%**
Sensitivity (*D* + *DCR* + *EILR* + *T*)	55.6%	**66.7%**	**66.7%**	**66.7%**	**66.7%**	55.6%
Specificity (*D*)	92.7%	97.6%	92.7%	**100%**	**100%**	92.7%
Specificity (*D* + *DCR* + *EILR* + *T*)	92.7%	97.6%	92.7%	97.6%	**100%**	97.6%
LHR+ (*D*)	4.56	0	0	//	//	**7.62**
LHR+ (*D* + *DCR* + *EILR* + *T*)	7.62	27.79	9.13	27.79	+∞	23.17
LHR− (*D*)	0.72	1.02	1.08	1	1	**0.48**
LHR- (*D* + *DCR* + *EILR* + *T*)	0.48	0.34	0.36	0.34	**0.33**	0.45

The symbol // indicates undefined. Best performances are marked in bold.

## 4 Discussion

In this work, we present a method for computing four local shape features on the ascending aorta and we compare the ability to identify patients at high risk of aneurysm growth through six classifiers based on these local parameters with the same classifiers based on the maximum diameter alone. We observed how each ML classifier returns a more accurate risk prediction when using the four shape features together.

The patients used for the shape metrics computation were derived from gated and non-gated acquisitions. We included a temporal filtering criterion of 6 months between the first and the second exam to reduce the uncertainty of the results, especially for the GR assessment (De Heer et al., 2011; Zubair et al., 2020). Moreover, we decided not to consider in this study patients with Marfan syndrome or in general congenital degenerative wall diseases since they could affect the results given the rapid aneurysm growth they could exhibit. The uncertainty introduced by using both CT-Scans and MRI-Angiographies is however mitigated by considering the selected exclusion criteria and by deriving segmented geometries corresponding to the intra-luminal regions from both types of acquisition. Minor and non-significant differences were indeed described when comparing diameters obtained from these two imaging techniques (Bons et al., 2019; Frazao et al., 2020).

After the segmentation and identification of the ascending aorta, the computation of shape metrics is performed on the entire AAo domain, including the Valsalva sinuses. This enables even aneurysms of the root region to be investigated. *D* is the only quantity that provides non-normalized local information with respect to the shape of the aorta. *DCR* returns a measurement of diameter in relation to a local length, which allows a better understanding of the differences between tall and robust patients and more slender individuals. *EILR*, on the other hand, gives information on the relationship between the external and the internal curvature line, to be carefully considered in the case of wall expansion toward the external direction of the aorta, as in saccular or root aneurysm (David, 2010). Finally, *T* returns another important piece of information, different from the previous ones, on how contorted and twisted the AAo is. Additional parameters such as volume and surface area of the ascending tract were not included as they were considered characteristic measures of vessel size and not shape (Heuts et al., 2020).

**FIGURE 6:**
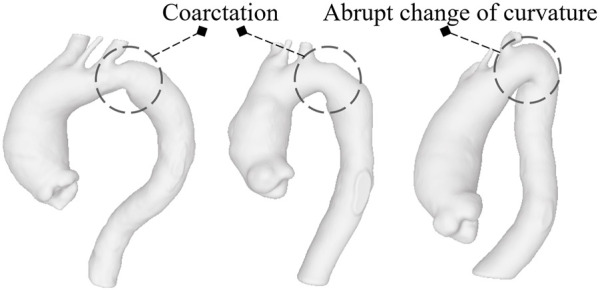
The models classified as FNs by SVM based on the four shapes features: the first two report aortic coarctation while the third shows a reduced radius of curvature between the arch and descending aorta.

We evaluate the growth rate as the difference between the maximum diameters derived from two exams, normalized by the distance in months between the two. This is, of course, a simplification since it seems that the aneurysm evolution follows an exponential law over time rather than a linear one (Hirose et al., 1995). In fact, according to Laplace’s law, wall tension is proportional to the vessel radius for a given blood pressure. So, growth rates become higher as the aorta progressively enlarges. Several empirical models have been presented to account for exponential growth rates exploiting multiple time exams (Gharahi et al., 2015). Information on how and whether aortic dilatation is accelerating over time could in fact further improve the prediction results. However, as most of the patients in the group had only two acquisitions and an exponential growth rate laws usually require controls over at least three different time instants for their validation (Martufi et al., 2013), the linear model was preferred. Additionally, in this study, *D* ^″^ and *D*′ can be at different centerline levels. Obviously, obtaining the maximum diameter at a certain distance from the annulus for the first exam and evaluating the diameter from the model related to the second acquisition using the same distance, without a new search for the maximum along the centerline, would produce different results in terms of GR with values that would certainly be lower or at most equal to those we used. As reflected in the data subdivision, only 9 (18%) patients showed a rapid evolution. This majority of patients with slow-growing aneurysms probably derives from the fact that the clinicians themselves, after a first 3-dimensional acquisition, often decide to make a second acquisition some months later when, based on clinical data, they suppose the disease will not evolve quickly and the dilatation phenomenon will not be abrupt and dangerous. It is worth noting that there are only 3 (6%) patients with the maximum diameter over 55 mm on whom local metrics were computed and two of these show a close to zero growth rate. We suppose they had not been surgically treated due to a precarious health state or because manual measurements of the diameter returned values below the threshold for surgery, really close to the value we measured. This, together with a correlation that is not strong neither statistically significant, are the reasons why classification based only on *D* results in failure. The performances of the individual diameter-based classifiers are, in fact, fairly low. This suggests that the diameter alone, current criterion for rupture risk, fails to accurately predict the growth, at least with respect to the data we collected. KNN, being based on the vote derived from the neighbouring classes, is the unique classifier able to discriminate at least 5 of the 9 patients of the high-GR risk class. Integrating the four features together and using the same KNN, a performance improvement (Table 2) is appreciable. However, among the classifiers based on all metrics, the best results are obtained using SVM. Although the sensitivity of SVM, i.e., the correct prediction rate for high-risk patients, never exceeds the 66.7% threshold, its accuracy, LHR+ and LHR− make it a good candidate in terms of utility (Ranganathan and Aggarwal, 2018). LHR + tending to +*∞* indicates that in case of a positive result, a patient definitely belongs to the high-risk group. LHR− = 0.33 means that a person on which a GR under the threshold is identified is about 3 (=1/0.33) times more likely to have a negative test than someone with a GR over the threshold. Since LHR+ and LHR− do not depend on the prevalence value, they are considered robust measures of the diagnostic capacity of the proposed classifiers. As reported in (Ray et al., 2010), an excellent classification method would return a LHR + higher than 10 and a LHR− lower than 0.1. Unfortunately, SVM fails to reach the threshold related to LHR−. However, we are confident that adding new parameters such as patient age, aortic valve type and possible related diseases, hypertension status or numerical simulation results (Groth et al., 2018; Biancolini et al., 2020) may improve the classification performances in that direction. Maier et al. (2010) have indeed proved how numerical simulation results as the stress at the wall and the ratio of wall stress and strength could be used to improve the prediction of abdominal aortic aneurysm rupture. The integration of this information may help to reduce the misclassifications that occur when using the shape features alone. Moreover, including detailed patient-specific material properties (Reeps et al., 2013) and an estimation of the geometry related to the stress-free state (Gee et al., 2009) could allow for even more accurate risk assessments. In any case, this is beyond the scope of this work, which concerns only the analysis of local parameters related to the ascending aorta shape. The presence of FNs in this type of classification is one of the most delicate aspects as it could lead to patients whose pathology is evolving quickly not being treated beforehand. In this regard, we consider interesting to observe the geometries related to the three patients classified as FNs from SVM for the full set of shape features, whose complete segmentations are shown in Figure 6. Two of them exhibit aortic coarctation (the only ones in the dataset) while one has an abrupt change between aortic arch and descending aorta with a very small radius of curvature. These morphological anomalies, altering the pressure gradient, obviously affect the fluid dynamics of the ascending aorta (Oliver et al., 2009). This could be the reason why, although the shape features of the ascending tract are not such as to characterize the patient as being at high risk, the disease undergoes a severe and rapid evolution over time. Four shape features alone are obviously not sufficient to predict the aneurysm growth but the results of this work clearly indicate the importance of considering the shape in studying the evolution of the pathology. Certainly, the AsAA surgical repair decision cannot derive exclusively from the analysis of the AAo alone but requires an integration of information from the upstream region (aortic valve) and the downstream part (arch and descending section). We also stress that the use of diameter as a criterion for surgery should not be replaced by these new features, but rather complemented by them since they, valuable in improving the assessment of the risk of growth, have not yet been proven effective in predicting the rupture. Overall, machine learning methods prove to be excellent candidates for improving the prediction of ascending aortic aneurysm shapes prone to rapid growth (Hahn et al., 2021) and delivering more personalized control and treatment plans (Monsalve-Torra et al., 2016). They are particularly suitable for integrating large amounts of data, including patient demographics, lifestyle factors, clinical history and medical images (Ashkezari et al., 2022). Moreover, ML algorithms can be used to track shape modifications in time and provide dynamic predictions of aortic aneurysm growth (Jiang et al., 2020), allowing for timely intervention. Unfortunately, multiple 3D longitudinal data are generally available for a limited number of patients and therefore a robust validation of the predictive ability is still challenging. Although this work shows that linking shape features derived from longitudinal data and ML classifiers is a promising approach to predict the aneurysm growth, some limitations need to be mentioned. The most important are the small dataset of patients used and the unequal distribution of classes. A more accurate study must necessarily include a larger number of patients with ECG-gated acquisitions performed at high resolution. A robust automatic segmentation method that removes manual correction processes would then be required, thus avoiding any bias introduced by the operator (Ashok and Gupta, 2021). Moreover, the uncertainty of the results is not only due to the segmentation task but also to the delimitation of the ascending aorta domain. Some of the patients were probably treated between one acquisition and the following with drugs such as beta-blockers that definitely affected the growth of the vessel over the months thus altering the correlation results between shape features and GR. It should also be mentioned that, as this study was exclusively related to the shape, we did not consider other important features such as material properties of the aorta wall and vessel thickness (Lin et al., 2022) which could further improve the results in terms of accuracy. The shape features described here relate exclusively to local properties. Future work will incorporate global shape features given by the modes of a statistical shape model (SSM) (Zheng et al., 2017) that will likely improve the model prediction. Although Cosentino et al. (2020) showed that, especially for the first principal modes, there is not a significant difference in terms of AAo wall shape between patients with bicuspid and tricuspid aortic valve, a subdivision into two different subsets would produce better results in terms of accuracy (Rooprai et al., 2019). Lastly, as previously introduced, it would also be interesting to assign a risk score to the valve type, to the possible level of calcification and to other co-existing risk factors and observe how the classification results would change according to them. A more in-depth analysis of the aortic valve would be consequently required although it is not straightforward to derive such information for all patients from this retrospective dataset. After further validations of the predictive capabilities through large-scale studies by including these multiple factors and overcoming the limitations described before, the reliability of these methods in clinical environments could be definitively established.

## 5 Conclusion

In this paper, we explain how to compute some shape features useful for classifying patients at high risk of rapid AsAA evolution. By using ML classifiers based on data derived from 50 patients, this work provides an indication that a set of AsAA local features could help in classifying the aneurysm growth potential more accurately than the maximum diameter alone. By treating large amounts of data, handling complex relationships and offering personalized predictions, machine learning can enhance the management and treatment of this dangerous disease. Deepening this combination of non-invasive geometric quantification and statistical machine learning methods and integrating these results with those derived from the numerical simulation could help in identifying aortic shapes potentially at risk of aneurysm growth and could certainly be useful not only for surgery planning but also for both the choice of therapy and the follow-up timing. In fact, this work might be important in a clinical environment to assess the risk of rupture of aneurysms during regular patient follow-up and might allow the development of personalized decision-making processes that will take into account not only the aneurysm shape but also several additional patient-specific data. The new shape features proposed here should not replace the diameter itself but complement it in order to have a more detailed understanding of this complex biological problem. It is obvious, therefore, that in order to consider these shape parameters as real biomarkers related to the AsAA evolution, the predictive capacity needs to be further strengthened by identifying and preparing a larger prospective study.

## Data Availability

The original contributions presented in the study are included in the article/supplementary materials, further inquiries can be directed to the corresponding author.
